# Climate and health education: A critical review at one medical school

**DOI:** 10.3389/fpubh.2022.1092359

**Published:** 2023-01-12

**Authors:** Lucy Greenwald, Olivia Blanchard, Colleen Hayden, Perry Sheffield

**Affiliations:** ^1^Department of Environmental Medicine and Public Health, Icahn School of Medicine at Mount Sinai, New York, NY, United States; ^2^The Leni and Peter W. May Department of Medical Education, Icahn School of Medicine at Mount Sinai, New York, NY, United States; ^3^Departments of Environmental Medicine, Public Health, and Pediatrics, Icahn School of Medicine at Mount Sinai, New York, NY, United States

**Keywords:** climate change, curriculum, education, medicine, curricular redesign

## Abstract

**Introduction:**

As medical schools continue to improve and refine their undergraduate curricula, they are also redefining the roadmap for preparing future generations of physicians. Climate change is a critical topic to integrate into medical education. This period of change for undergraduate medical education coincides with a surge in interest and design efforts for climate and health curricula in health professional education, but this nascent field has yet to be solidly institutionalized. To continue to grow the number of medical students who achieve competency in the effects of climate change on individual health and the health of the planet during their training, we must examine what has worked to date and continue to shift our approach as curricular changes are implemented for feasibility and relevancy.

**Objective and methods:**

In the present study, we assessed the “climate and health” content at one northeastern U.S. medical school that is undergoing an overhaul of their entire curriculum to explore strategies to deliver more robust climate health education in the context of the educational redesign. We conducted 1) a retrospective review of the now four-year-old initiative to investigate the sustainability of the original content, and 2) semi-structured interviews with lecturers, course directors, and medical education coordinators involved in implementation, and with faculty tasked with developing the upcoming curricular redesign.

**Results and discussion:**

Of the original implementation plan, the content was still present in nine of the 14 lectures. Themes determined from our conversations with involved faculty included the need for 1) a shared vision throughout the content arc, 2) further professional development for faculty, and 3) involvement of summative assessment for students and the content itself to ensure longevity. The interviews also highlighted the importance of developing climate-specific resources that fit within the school's new curricular priorities. This critical review can serve as a case study in curriculum to inform other schools undergoing similar changes.

## 1. Introduction

The overarching objective of undergraduate medical education curricula is to provide students with the scientific knowledge and practical skills to be accomplished and responsible physicians (1). Historically, medical school curricula undergo frequent content, format, and faculty changes as well as periodic large scale reorganization (2), which is currently happening across the country (3). Prominent trends throughout the present curricular developments include condensing the early coursework and introducing more content on social science and policy (4) and structural determinants of health (5, 6). The driving forces for these changes include Association of American Medical Colleges (AAMC), Liaison Committee on Medical Education (LCME), and United States Medical Licensing Evaluation (USMLE) pressures and student demand (7–13).

Climate change is one of the large societal issues being integrated into some medical school curricula. Climate change has been on the radar of the general public for years but has only slowly gained traction as a political, social, and medical crisis. The effects of climate change on health come from both the indirect impacts of exacerbating inequities in SDOH, with the earliest and most prominent effects of climate change affecting those in low income and disadvantaged communities (14), and the direct impacts of heat, extreme weather events, pollution, wildfires, and other phenomena. Infants and young children, older adults, and people with disabilities are also among the most vulnerable to the effects of climate change. A breadth of research shows the direct clinical impacts of climate change in all medical disciplines [cardiac health, (15); pulmonary health, (16); renal health, (17); infectious disease, (18, 19); psychiatry, (20); emergency medicine, (21); pediatrics, (22, 23); gynecology, (24)], but this research has not translated to inclusion into medical school curricula at the same rate. In a survey conducted by the International Federation of Medical Students associations, only 15% of the 2,817 medical schools included climate change in their curricula (25). In recent years, groups within medical schools have worked to build and adapt curricular initiatives that reflect the nature of climate change as a societal issue and a direct threat to health. Various methods for implementation have been adapted: Emory University and the Icahn School of Medicine at Mount Sinai (ISMMS) have adopted a disseminated design with climate change and health content spread throughout pre-clerkship courses and small group discussions (26), Queen's University Belfast, Stanford, and UC-Berkeley UCSF Joint Medical Program have an elective-based approach, and Georgetown School of Medicine and Harvard Medical School offer clinical scenario exercises to expose students to the practical applications of climate change (27). Because climate change results in pervasive, universal, and ever worsening health problems, it remains crucial to educate the students who will be responsible for human health on its impacts.

**Figure 1:**
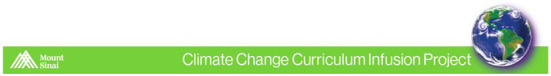
The CCCIP banner that appeared on each of the pre-prepared slides (26).

We are in a critical period for understanding curricular initiatives in climate change and health to ensure their sustainability. The ISMMS MD program is undergoing a curricular reform across all facets of the educational program. The climate change curriculum infusion project (CCCIP) is the initiative that has coordinated the introduction of climate change content at ISMMS since 2018. The student-led, faculty supported group responsible for the inception of the project designed stand-alone slides, each with a recognizable banner (Figure 1), to be incorporated in 14 lectures across six courses in the first 2 years of the pre-clerkship curriculum (26). Two rounds of student feedback (*n* = 74) of the CCCIP concluded that the content was appropriate in the courses (88%) and important to their medical education (83%). The feedback also indicated that students did not remember the content well (78%) and that the climate-related content at ISMMS did not match their expectations [62%; (26)].

The goal of the present study is to explore a nationally relevant case study of the ISMMS' climate change content as it relates to a drastic curricular redesign. We aim to assess the CCCIP implementation from the perspective of the ISMMS faculty, understand the challenges to implementing the content as presented, and assess ways to improve the success and sustainability of the information in the new conceptual framework.

## 2. Methods

### 2.1. Retrospective review of CCCIP

#### 2.1.1. Study design and data collection

The first component of this study was a retrospective review of CCCIP content continuity. We identified the lectures where CCCIP content was originally accepted by course directors and lecturers by following the CCCIP records (26) from the inception of the program. Medical administrators at ISMMS granted us access to the *Blackboard* course websites for all courses from the 2020–2021 and 2021–2022 academic years identified to have lectures with CCCIP material. With the timetabled lectures from 2018 as a guide, these courses were systematically reviewed to identify where CCCIP content was still being used.

#### 2.1.2. Data analysis

Lectures where we found slides with the CCCIP banner were counted as lectures where the content was still present. The data for both years of content were recorded and helped to inform the second component of the study.

### 2.2. Assessment of faculty experience

#### 2.2.1. Study design and data collection

The second component of the study aimed to gather faculty feedback on the CCCIP. To better understand faculty experience with CCCIP implementation, a mixed methods interview-based exploratory study was designed. The study was deemed exempt by the Mount Sinai Institutional Review Board (IRB). Inclusion criteria were based on participation in the original CCCIP. Eligible faculty members included lecturers who were tasked with delivering CCCIP content, course directors for courses where CCCIP content was included, and medical education leadership. Two separate semi-structured interview guides were created, one for lecturers and course directors directly involved in the CCCIP and one for medical education faculty who had knowledge of the aims of the content implementation and who are involved in the current curriculum redesign. The guides were designed by consulting studies with similar lenses of curriculum implementation (10, 28–30) and by reviewing literature on qualitative research methods (31).

Eligible faculty members (nine lecturers, of whom five are also course directors, and six leaders in medical education) were emailed with information about the study, the research information sheet, and a request to schedule a 30-min interview. Once a time slot was selected, a calendar invitation was sent to the faculty member with a HIPAA-compliant videoconference *Zoom* link. Zoom sessions were run by one interview lead and notes were taken concurrently by another researcher. After obtaining consent, each session was recorded for note-taking purposes. Interview questions included three introductory questions related to the interviewee's field of practice, ten baseline questions regarding lecture content and delivery for lecturers and course directors, and seven baseline questions regarding curriculum design and sustainability for medical education faculty and those involved in the curricular redesign team (Table 1). Following the conclusion of the interviews, the recordings were reviewed by the research lead to supplement the notes, as needed. Once the final data were organized, recordings were permanently discarded and data were stripped of all identifiers.

**Table 1:** Closed and open interview questions for the semi-structured interviews for the climate content evaluation.

**Introductory questions**	
	What is your field of practice—clinical practice and/or medical education focus?
	Do you feel that Climate Change is important in your field of medicine?
	*Follow-up: How important (1–5, 5 being critically important)*
	Is your specialty addressing climate change as a health issue?
**Baseline questions for lecturers/course directors**	
	Was the CCCIP information integrated in your lecture/s?
	*Follow-up: Did the CCCIP slides feel like a natural fit with your existing slides?*
	Did the CCCIP information come up in any other part of the course?
	Do you plan to continue to include the CCCIP information in your lecture/s?
	How comfortable were you in teaching the climate change content in your course? (1–5, 5 being very comfortable)
	*Follow-up: Are there steps we can take to help faculty feel more empowered to teach this aspect of the curriculum?*
	Do you believe that the students engaged with this aspect of the course content?
	Did you find anything particularly helpful in implementing this content?
	Did you face any challenges when implementing this content?
	How can we better support you in successfully implementing climate change in medicine material?
	With the upcoming curriculum redesign, do you see a place for cross-cutting topic threads like climate change and other SDOH?
	Do you have any further ideas for more successful implementation of this information?
**Baseline questions for medical education faculty**	
	What was your role in implementation of the CCCIP project?
	From your view in medical education, do you believe that the lectures given have impacted the way that the students view the impacts of climate change in medicine?
	How can we help our faculty to feel empowered to teach this content?
	Do you feel like there is room and opportunity to improve the CCCIP?
	What would you say, if any, are the institutional barriers to creating and implementing thematic course content across multiple courses?
	How can we approach sustainability of the course content delivery as lecturers and course directors may change?
	With the upcoming curriculum redesign, do you see a place for cross-cutting topic threads like climate change and other SDOH?

#### 2.2.2. Data analysis

Interviews were reviewed and characterized throughout data collection. Qualitative interview data were coded by a single coder using an inductive approach (32). During analysis of individual interview transcripts, ideas in each interview were noted and subsequently added to a separate spreadsheet. The same spreadsheet was used to organize ideas from every interview and served as an initial code-book. Analyses were checked by a second, independent coder. The additional coder chose three interviews to code at random, after which the two coders reviewed the independently generated codes for consistency. Once all interviews were coded, results were refined and synthesized into broader thematic determinations. Quantitative, Likert-style questions were assessed using parametric summary statistics.

## 3. Results

### 3.1. Retrospective review of CCCIP content

In the CCCIP, content was initially (2018) planned for a total of 14 lectures across 6 courses (26). In the retrospective review of these lectures from 2020 to 2022, we found that the content was present in nine lectures (64%) across five courses (Table 2). The number and content of CCCIP lecture slides used in each lecture changed from year to year depending on lecturer preference. Interviews with course faculty revealed that CCCIP content was implemented in one lecture not originally included (Alzheimer's disease, *Brain and Behavior Course*). Content that was originally planned for another lecture (asthma, *Pulmonary Pathophysiology course*) was used initially, but was removed prior to the 2020–2021 academic year and therefore not included in this review.

**Table 2:** Results of the retrospective (2020–2022) review of CCCIP inclusion, presented in chronological order of content delivery through the 2 years of the pre-clerkship curriculum.

**Course**	**Lecture topic for which the CCCIP was planned**	**School year of lecture presentation**	**CCCIP slides present in 2020–2021?**	**CCCIP slides present in 2021–2022?**
**The Art and Science of Medicine**				
	Course introduction	Year 1	Yes, as a separate resource	Yes
	Obtaining an effective social history	Year 1	Yes	Yes
	Social determinants of health	Year 1	No	No
**Immunology**				
	Immunology of allergic responses	Year 1	Yes	Yes
**Medical Microbiology**				
	Bacterial biology and mechanisms	Year 1	Yes	Yes
	Bacterial GI pathogens	Year 1	Yes	Yes
	Vector-borne and zoonotic bacterial infections	Year 1	Yes	Yes
	Viral vector-borne infections and zoonoses	Year 1	Yes	Yes
	Global perspective	Year 1	Yes	Yes
**Brain and Behavior: Neurology, Neuroanatomy, and Psychiatry**				
	Child development	Year 2	No	No
	ADHD and autism	Year 2	No	No
	Nutritional and metabolic disorders of the CNS	Year 2	Lecture not given	Lecture not given
	Alzheimer's disease^*^	Year 2	Yes	Yes
**Pulmonary Pathophysiology**				
	Asthma	Year 2	No	No
**Cardiovascular Pathophysiology**				
	Cardiovascular disorders	Year 2	Lecture not given	Lecture not given

* CCCIP content for the Alzheimer's disease lecture was not originally reported in Kligler et al. (26), but was identified as a lecture with Information about the lecture topics having CCCIP lecture slides prepared for them is taken from Kligler et al. (26). This review investigated the presence of these lectures in the 2020–2021 and 2021–2022 curricula. CCCIP content by institutional memory and verified in interviews with course faculty.

### 3.2. Assessment of faculty experience

Interviews were conducted with seven of the nine recruited lecturers (including four of the five course directors) and with two of the six faculty members in medical education leadership. Faculty members were given unique identifiers A-I. The semi-structured interviews revealed several common ideas that were then organized into three major thematic umbrellas with regard to ensuring sustainable content development: (1) the necessity of centralization and a shared vision; (2) adequate professional development; and (3) assessment of student learning and of the content itself (Table 3). Coding comparisons revealed high inter-rater reliability. Barriers to general curriculum development and re-design had a high degree of consistency with those felt by the faculty involved in the CCCIP.

**Table 3:** Codebook from faculty interviews with lecturers and course directors.

Identified theme^*^	**Definition**	**Responses comprising the thematic designations: enablers of implementation**	**Responses comprising the thematic designation: challenges to implementation**
Shared vision	The ability for all contributors and participants to see and understand the full arc of the content, including learning objectives, location of content, and assessment milestones.		Lack of centralized content arc
Faculty development	Educational support for faculty in increasing their ability to successfully deliver necessary content.	Faculty interest in climate change: personal reasons	Lack of faculty expertise
		Faculty interest in climate change: visibility during clinical practice	Fit of CCCIP content: forced
		Fit of CCCIP content: natural	
		Drivers for implementation: student involvement	
		Drivers for implementation: faculty buy-in	
Assessment	Measurable results of both student learning, in the form of summative assessment, and the content implementation, in the form of satisfying institutional or accreditation requirements.		Lack of summative assessment
			Lack of time and space in the curriculum

* Responses throughout interviews that touched on similar concepts were grouped together in “Identified themes.”

#### 3.2.1. Shared vision

The most commonly cited challenge was the lack of centralization in terms of the organization of the content arc and access of the contributors and participants to the full plan. When the CCCIP began, permission was granted from course directors to include the slides into their course. Slides were given to individual lecturers to integrate into their existing content, but participants noted a lack of knowledge of the “bigger picture.” Several faculty expressed the need for more visible leadership as well as an overt curricular map to provide context and to motivate them to present the material in a meaningful way. For example, one lecturer/course director (study participant C) noted that they “never heard if the content was implemented in other courses” and another lecturer (study participant E) thought that knowing what had been taught so far would make it easier to contextualize their piece of the curricular thread in relation to what had been taught about the topic in previous courses.

Themes discerned from conversations with faculty specifically involved in the upcoming curriculum redesign echoed similar themes to the lecturers and course directors. They further highlighted the need for comprehensive resources for proposed curriculum enhancements, with designs that involve a full educational arc:

“It's so critical that we have a curriculum map and an inventory of where [the content] is taught and where it is assessed. It needs to be big picture: What's the arc? Where do we start from? And Where are we going?…Do folks have learning objectives throughout? Do we have assessments? Are there questions on any exams related to this? This is a very important database of information to have as you think through the curriculum going forward… Where is it actually meaningful?... It really is figuring out how do we ensure the long term retention of it for the students.”

- Medical education leadership (study participant I).

#### 3.2.2. Faculty development

All but one of the interviewed faculty members agreed that climate change is important in their field, and in medicine in general (average = 3.75 on 5-point Likert scale; SD = 1.0206, median = 3.75). Reasons for this importance ranged from direct impacts on patient health, such as weather events impacting the ability of patients to receive care, to indirect impacts involving SDOH, with one lecturer/course director (study participant A) noting “people's social circumstances greatly affect whether they need intensive care.” Those that described lower degrees of importance of climate change in their field noted that, to their knowledge, the question of its impact had not yet been addressed. Lecturers had various reasons for agreeing to include information about climate change and health in their material. Lecturers with connections to climate change outside of the CCCIP generally felt more comfort in developing the material. Some faculty had a personal interest in climate change: one lecturer/course director (study participant F) cited family members who work directly in the field and act as climate change activists while another lecturer (study participant B) cited personal fears about the climate crisis outside of their occupation. Other faculty became invested in the health impacts of climate change through seeing it in their work. For example, one lecturer explained

“I think …in my education [climate change] didn't play any role, so I think it was really when I was working on the ground and I was seeing the effect…I was seeing malaria epidemics were happening where, according to the books, they shouldn't have happened…[Climate change became important] when I really had contact with it and really saw the consequences.”

Lecturer (study participant D).

The role of students came up as an important topic throughout the interviews. One lecturer/course director (study participant C) noted that students in this generation “are more attuned to and more concerned with these issues,” making climate change a comfortable and important topic to bring into lectures. The idea of students as drivers of content development was consistent throughout almost every interview. Many faculty cited the CCCIP initiative as essential in reminding faculty that these topics are important. One lecturer (study participant D) stated that “what you are doing is like lobbying, you just have to continue lobbying” and another lecturer (study participant B) noted that being brought the material by the research group was the first time they had thought about climate change as it relates to their field. Interviewees also identified buy-in from medical education faculty as an essential driver for content development and reform. Some faculty participants explained that support from higher level administrators would make them feel that the new content is necessary, that there is a network of support, and that their labor involved in curricular development is valued.

Comfort and expertise with climate change and health was variable across the lecturers (average = 3.50 on 5-point Likert scale; SD = 1.643, median = 4.0). Limited faculty development and time were noted as a substantial challenge for those who were less comfortable with the topic itself, noting a lack of “bandwidth in the midst of the course to incorporate new material” and that there was “no support, no one in charge was giving a presentation” (lecturer, study participant E) during the CCCIP. Expansion of faculty development around climate change and SDOH through experiences such as an educational development session, written faculty guide, annual event with expert speakers, or a learning module for faculty were cited as ways to improve faculty comfort.

The challenge of faculty expertise on climate change was also identified as a factor in feedback on the efficacy of the pre-made CCCIP slides. While about half of faculty members felt that the CCCIP information fit “very well” into their existing course material, others noted that the slides felt “a little disjointed” or “like a post-script for the lecture rather than something that nicely tied it together” (lecturer/course director, study participant G). Positive attributes of the slides themselves included the recognizability of the banner (Figure 1) and the clarity of having info-graphic style slides. Faculty members had differing opinions about whether having the pre-made slides was helpful in incorporating the new information, or if providing the slides was a barrier to feeling ownership and confidence in the material. One lecturer (study participant E) believed that the pre-made slides were helpful noting that if we “had not given [them] the slides, [they] probably would not have included it” but that the ease of having slides allowed them to avoid exploring the topic further and including the concept in their own words, making the slide more of a “shortsighted solution.” Overall, faculty motivation appears to be heterogeneous with lack of personal education as a substantial barrier to successful and motivated implementation.

#### 3.2.3. Assessment

Assessment is a reflection of both student learning, in the form of summative assessment, and of the content implementation, in the form of institutional or accreditation requirements. Having these quantifiable assessments increases the pressure on institutions to include curricular topics and increases the pressure on students to internalize the content. The need for these outcomes in the success of any curriculum is clear when considering the frequently cited challenge of time and space constraints in medical education. One lecturer (study participant D) explained this challenge, saying “I think the problem is that medicine is always growing, but the time we have face to face with students never grows.” Faculty on the medical education leadership team (study participant H) echoed this idea with the notion that “there are many topics that people are passionate about, but [when something is added] something has to come out.” The upcoming curriculum reform plan for ISMMS includes changes to the mode of instruction, moving away from lecture-based learning toward more engaged learning modalities, which anticipates all courses having to confront the challenge of curricular space and prioritization:

“Everybody is going to have to pull out what's been most critical… we are going to have to figure out how we fit those into small group discussions and case-based discussions…[we have to figure out if there] is stuff with climate change that is …self-taught that we can still require [and] assess, but …in a way where the students are not going to gloss over it.”

- Medical education leadership (study participant I).

Guidelines in the form of institutional requirements and summative assessment shape what continues to be included in medical education. None of the CCCIP information was included in course assessments to date. Several faculty members noted lack of assessment both at the school and the USMLE level as barriers to advocating for further development in this content area. Most faculty agreed that assessment is an important tool for learning. One lecturer/course director (study participant C) stated, “assessments should reflect what we think is most important for students to learn and to understand and I think that if we are not assessing that content that that's sending a message that it is maybe not that important.” In terms of a message of importance coming from governing bodies of medical education, faculty cited the student and educator fixation on the NBME boards to dictate was content is emphasized:

“There's so much major biomedical content that you have to have to get you ready for step 1 and the clinicals…a lot of this other [material]... the touchy-feely side of medicine…gets lost a bit.”

- Medical education leadership (study participant I).

“we're not going to get any points for it…for accreditation because we're not assessing it. We're just saying we did something, but we really didn't do it. We didn't go through it in a meaningful way.”

- Medical education leadership (study participant I).

## 4. Discussion

The results of the retrospective review portion of this study show the longevity of the prior climate content integration at ISMMS, and the qualitative interview portion of this study serves to help cultivate an understanding of the reasons behind its mixed successes and failures. A majority of the CCCIP content that was created in 2018 was carried through to the 2021–2022 curriculum, but not all of it. This is consistent with the changing nature of curriculum and educational priorities (2). Two interesting changes to the pre-made CCCIP content noted in the interviews were 1) the removal from one lecture after having been in place the years prior, and 2) the addition of content to a lecture where it was not originally planned to be. First considerations of the removal of the content from a lecture may suggest that the content was deemed unimportant, but based on our conversations with lecturers, it may more likely reflect discomfort with the material and curricular time constraints. In a survey of 84 international health professional schools and programs, 71% of respondents indicated that they encountered challenges to instituting climate change content in their curriculum, with 24% indicating lack of teaching materials and expertise and 29% indicating no available space in the core curriculum, similar to challenges identified in our conversations (30). On the other hand, addition of climate related content into an unplanned lecture may point to a sustainable impact of the CCCIP initiative on the faculty themselves. Teaching and learning are often thought to be intertwined processes that happen simultaneously and symbiotically (33). Teaching the CCCIP material may in turn serve to make the lecturer more aware and interested in the topic. This is consistent with the idea that the CCCIP initiative and the student involvement in the curriculum helped to drive content implementation that was present across multiple interviews. The idea of student lead initiatives as drivers of change in medical education is present both in institutions initiating climate change education (34–36) and across additional domains of educational reform, outreach services, and advocacy groups as students engage in extracurricular activities and research throughout their medical education (8, 37, 38). Student-faculty partnerships are integral to the development and sustainability of curricular changes and accountability in the health sector (36). While students may be able to take on some of the required work in facilitating learning, including creating learning materials, teaching their peers, and leading faculty development, the investment of faculty support, clinical expertise, and status in the institution is continuously necessary for the longevity of the initiative (35). When medical education and faculty embrace students as partners, they are able to become more invested in shaping their own education.

**Figure 2:**
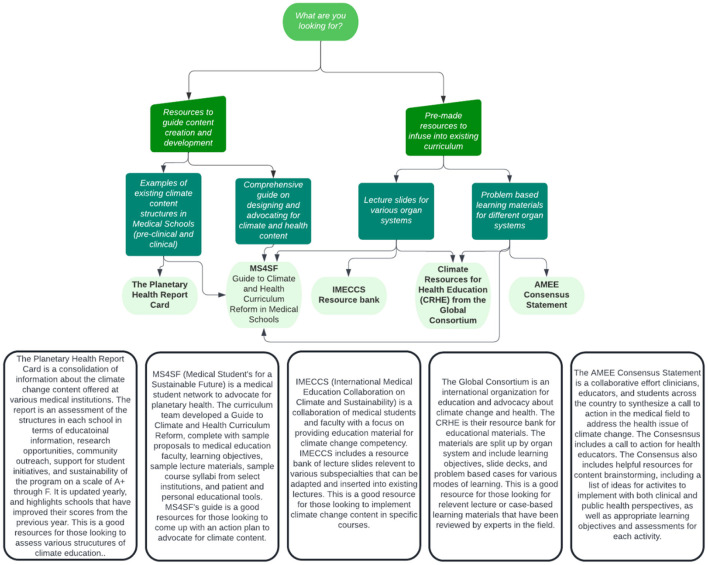
A guide for the various available resources for climate change content development and implementation [graphic created by Lucy Greenwald with information sourced from https://phreportcard.org/, https://ms4sf.org/, https://www.imeccs.org/, https://climatehealthed.org/, and (39)].

Active collaborations to organize student and faculty advocacy efforts can aid in the creation and development of future curricula at ISMMS and other institutions. International organizations (International Medical Education Collaboration on Climate and Sustainability, IMECCS; and the Global Consortium on Climate and Health Education, GCCHE), international initiatives (Planetary Health Report Card, PHRC; and the Association of Medical Education in Europe's Consensus Statement, AMEE), and national student networks (Medical Students for a Sustainable Future, MS4SF) offer extensive and overlapping resources for guidance on advocating for inclusion of content, content development, and an in-depth content repository of content (summarized in Figure 2).

Three key barriers to successful and sustainable content integration in the CCCIP arose from conversations with faculty: Lack of a shared vision for the content arc, inadequate faculty development, and failure to incorporate assessment. These essential elements of content design were echoed by the medical education faculty preparing to implement the new curriculum. The overall challenges faced by the ISMMS faculty in implementing the CCCIP curriculum match those seen in other institutions (30). When looking across institutions, it is clear that the efforts to improve an institution's climate literacy is never without its challenges. The PHRC provides an interesting look into the relative efficacy of climate curricula and additional aspects of sustainability and climate consciousness at different institutions. The results of the first year (2019–2020) of the PHRC indicated that zero out of the 13 participating institutions received an “A” grade (80% of possible points) and the results of the second year (2020–2021) indicated that only one institution, Emory University (Atlanta, GA, USA) out of 62 medical schools in five countries received an “A-” (40). These results indicate that significant improvement is still needed across all participating institutions (41).

Overall, we found that a transparent and intentional approach to implementation involving accessible content mapping, faculty education, and formal assessment of related content may help to improve the overall knowledge base of the institution and its students. These findings are consistent with the some of the important points in the six-step approach to curriculum development in medical education. The six-steps include “performing a needs assessment, determining and prioritizing content, writing goals and objectives, selecting teaching/educational strategies, implementation of the curriculum, and evaluation and application of lessons learned” (42, 43). Without clear content mapping with a shared vision, faculty development, and formal assessment, these steps cannot be met.

Following the upcoming shift away from primarily lecture-based education, aspects of how content is best delivered at ISMMS, including climate change and health education, may need to be re-thought. As this research explores the CCCIP at ISMMS as it relates to the imminent curricular redesign, it can have national relevance as a case study for other medical schools. Institutions aiming to integrate climate and health education, and advocacy groups with hopes of empowering their institutions to do so, must be able to develop and promote these content initiatives in the context of wider curriculum development.

LCME guidelines create unique opportunities for climate change and other topics surrounding SDOH to provide enhancement of real-world applications of the scientific basis of medical education (13). With these guidelines, there has been a growing interest in teaching SDOH in medical education (44), a change that can both serve to highlight the importance of these issues in health and health inequity, and help to fulfill the accreditation requirements of the institution. While climate change impacts and exacerbates existing inequities of SDOH (*What is Climate Change?*), the reality of climate change as a present and imminent threat to the health and lives of the population may be better stressed by separating it from SDOH and focusing on ecologic determinants of health, such as air and ocean pollution, global warming, and declining biodiversity (45). This approach is also more holistic in examining the impact of the health of the planet on human and community wellbeing at a systems level, including more comprehensive factors, such as “ecological, social, cultural, and intergenerational determinants of health” and encouraging participation of community, policy, and indigenous programs outside of the health sector to inform perspectives (45). As the emphasis of medical curricula shifts to highlight the patient in context, individuals' social and physical environments play an even larger role in health (46).

At ISMMS, the LCME guided “societal problems” will be integrated in six threads throughout the curricular arc. These threads have already been chosen as an extension of the named priorities of the institution: Scholarly Discovery, Advocacy, Social Justice and Anti-Oppression, Healthcare Delivery Science, Medical Decision-Making, and Leadership and Professional Identity Formation. Climate change is included under the umbrella of “Advocacy, Social Justice, and Anti-Oppression.” Some possibilities for the future of the CCCIP include a pre-clerkship informal extra-curricular elective, a clerkship elective course, generating fully developed problem-based learning cases to be integrated in pre-curriculum courses, and continued advocacy for climate literacy of all faculty at the institution, integrating faculty development across subspecialties.

### 4.1. Limitations

The major limitation of this study was that we reviewed only one medical school's climate content. Additionally, we only reviewed the content from faculty involved in the first 2 years of the pre-clinical curriculum. Additional institutions and inclusion of faculty with greater diversity of educator experiences of climate content would be needed to make the conclusions generalizable to the public. Nevertheless, we were able to have meaningful conversations with faculty at each level of leadership in the curriculum that provided valuable information to consider.

## 5. Conclusions

From the retrospective review and qualitative interviews with faculty involved with delivering climate change content, we identified key steps that are needed to implement successful and sustainable curricula. It is necessary to stay active and continue to build fully realized curricula with the help of available resources, especially in the current period of reviewing and revitalizing medical education. Advocates must engage medical education deans and faculty to assure that there is higher-level understanding of the importance of this education. Further advocacy must extend beyond the institutional level to national networks of decision makers in medical education standards (USMLE, LCME, and AAMC). Climate and health literacy must be on the radar of all those with the power to make curricular decisions for the benefit of all current and future physicians and patients. Providers have direct access to communities, and therefore unique opportunities to recognize climate change and prepare patients for its effects. As respected members of society who are first-hand witnesses to the effects of the crisis, physicians must take active roles in preventing its worst effects by advocating for more robust climate action—specifically reducing healthcare sector carbon emissions and building climate resilient health systems. As a society we have begun to become numb to the devastating effects of catastrophes that we encounter every day (47). We must remember that climate change is here, it is impacting our health, and it is accelerating.

## Data availability statement

The original contributions presented in the study are included in the article, further inquiries can be directed to the corresponding author.

## Ethics statement

Studies involving human participants are reviewed and approved by the Icahn School of Medicine at Mount Sinai IRB. This study was reviewed by the IRB and deemed exempt. The participants were provided the study information and gave verbal consent prior to the start of the interviews.

## Author contributions

LG, OB, and PS conceived of the research idea and participated in data collection and analysis. LG wrote the manuscript with supervision from PS and editing from OB. CH provided expertise in LCME and accreditation requirements and reviewed and edited the manuscript. All authors discussed the results and helped shape the final product.
